# Changes in technological innovation efficiency and influencing factors of listed textile and apparel companies research——Based on three-stage DEA with Tobit modeling

**DOI:** 10.1371/journal.pone.0307820

**Published:** 2024-08-08

**Authors:** Guang Chen, Fei Chen

**Affiliations:** 1 School of Textile Science and Engineering, Zhejiang Sci-Tech University, Hangzhou, China; 2 School of Economics and Management, Zhejiang Sci-Tech University, Hangzhou, China; Guilin University of Aerospace Technology, CHINA

## Abstract

The key to high-quality development in the textile and apparel industry lies in enhancing technological innovation and optimizing the efficiency of technological innovation. Based on data from 60 A-share listed companies in the textile and apparel sector in China from 2013 to 2022, this study employs a three-stage DEA model and the Malmquist index model to measure changes in technological innovation efficiency from static and dynamic perspectives. Additionally, it uses a Tobit model to analyze the impact and mechanisms of management and financial factors on technological innovation efficiency. The results indicate that: (1) Compared to the manufacturing industry and its sub-sectors, the overall technological innovation efficiency of listed textile and apparel companies was relatively low and showed a declining trend between 2013 and 2022; (2) Over the decade, the average total factor productivity of these listed companies increased by 1.7%, exhibiting a "W" shaped fluctuation, with technological progress, pure technical efficiency, and scale efficiency all showing weak improvement; (3) Management and financial factors significantly influence technological innovation efficiency. Specifically, employee quality, profitability, and operational capability are positively correlated with technological innovation efficiency and have long-term effectiveness, while firm age, management costs, equity concentration, development ability, and debt repayment capacity are negatively correlated with technological innovation efficiency; (4) Different types of enterprises show differences in the significance of management factors, while whether the same person holds both managerial positions significantly affects financial factors.

## 1 Introduction

As a historically beneficial sector of the Chinese economy, the textile and apparel industry has a significant impact on employment, commerce and export, technological innovation, and other areas in addition to the country’s economic development [1]. Since the abolition of the quota system for textile and apparel products in 2005, China’s textile and apparel industry has seized new opportunities for expansion, the market development potential has been further stimulated, and international competitiveness has been increasingly improved [2]. By the end of 2022, enterprises above the scale of China’s textile and garment industry reached 34,000, the average number of workers in the industry was 4,954,000, the total assets amounted to 332.808 billion Yuan, and the cumulative total profit realized was 161.98 billion Yuan, which accounted for 7.2%, 6.4%, 2.1%, and 1.9% of the whole industrial industry, respectively [3].

In the development process of the new era, technological innovation and optimization the efficiency of technological innovation have become one of the core paths to promote the high-quality development of China’s industrial economy [4]. The key to the high-quality development of the textile and garment industry also lies in technological innovation, which can not only bring about the continuous improvement of products and processes, but also effectively enhance the rate of added value of products. In order to effectively guide China’s textile and apparel industry to achieve high-quality development, the state has issued a number of measures to provide policy protection. In April 2022, the Ministry of Industry and Information Technology and the National Development and Reform Commission jointly issued the "Guiding Opinions on High-Quality Development of the Industrial Textiles Industry", and in August 2023, the China Textile Industry Federation issued the "Action Outline for Building a Modernized Textile Industrial System (2022–2035)". Efficiency is the primary metric used to assess the textile and apparel industry’s capacity for technological innovation. This research has significant theoretical and practical implications for advancing the high-quality development of the textile and apparel industry. It also helps to identify ways to improve technological innovation efficiency, prevent resource duplication and decentralization, and accurately identify the critical factors influencing technological innovation efficiency.

**Fig 1:**
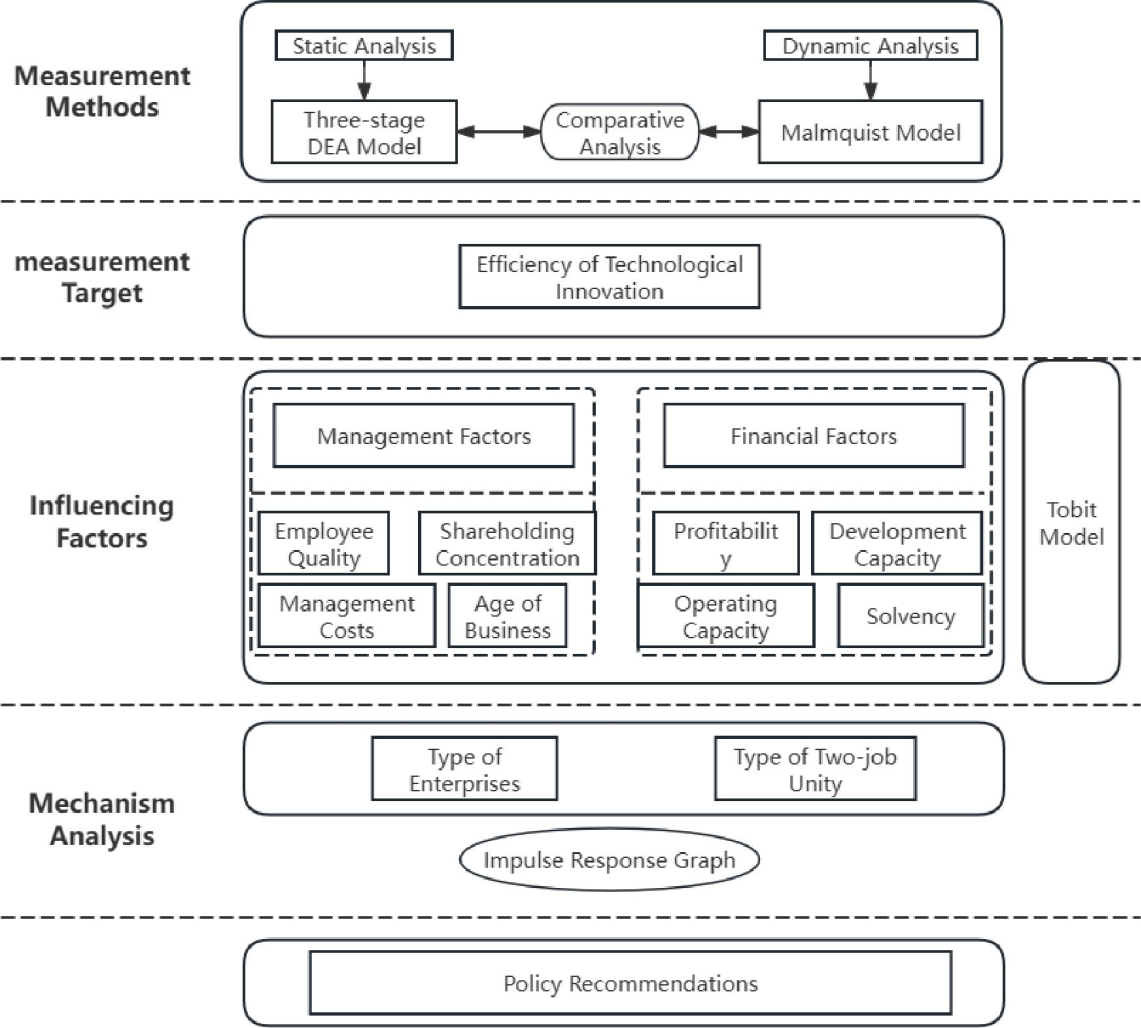
The research framework of this paper.

Currently, what is the status of technological innovation efficiency in China’s textile and apparel industry? How can it be accurately measured? What factors influence technological innovation efficiency, and how do these mechanisms operate? These problems still require in-depth investigation and analysis. In order to precisely assess the technological innovation efficiency of China’s textile and clothing sector, investigate the variables that impact this efficiency, uncover the workings of its operation, and subsequently offer a specific point of reference for the formulation of government policy. As the research object, this paper examines the technological innovation efficiency of China’s listed textile and garment companies from both static and dynamic viewpoints. It then looks for the factors that influence the enterprise’s technological innovation efficiency, illuminating its internal relationships and workings. The research framework of this paper is as shown in Fig 1.

### 2 Literature review

There is some complexity involved in evaluating technological innovation efficiency, and the majority of research that has been done so far has focused on how to assess an enterprise’s technological innovation efficiency from the perspective of changing the evaluation process, investigating internal enterprise factors, managing external environment factors, etc. However, a standard index system and evaluation methodology have not yet been developed.

The primary research can be categorized into two groups based on how technological innovation efficiency is evaluated: the parametric approach, which is represented by Stochastic Frontier Analysis (SFA) [5,6], and the non-parametric method, which is represented by Data Envelopment Analysis (DEA) [7,8]. Wu [9] used SFA to empirically investigate the technical efficiency of 603 family farms and the factors influencing it. Huang et al. [10] assessed total factor energy productivity based on the stochastic frontier analysis (SFA) method and analyzed the impact of industrial intelligence on energy productivity using Chinese manufacturing panel data. Yigiteli and Sanli [11] used the panel stochastic frontier approach (SFA) to analyze the total factor productivity (TFP) growth of 26 regions in Turkey from 2004 to 2020. Zhang and Chen [12] analyzed the innovation and entrepreneurship efficiency of China’s crowdsourcing spaces using a three-stage DEA modeling approach. Based on the effective exclusion of external environmental variables like government subsidies, external financing, and service personnel, the authors examined the differences between provinces. Raj et al.[13] used a three-stage closed system fuzzy network data envelopment analysis (DEA) to assess the capabilities of reinsurance companies operating in India by decomposing a complex service process into three sub-processes. Kang [14] used data envelopment analysis (DEA) and DEA windowing to assess the performance of provincial road safety in China from 2012 to 2021 from the input-output perspective of road safety risk. Han et al. [15] investigated the impact of CEO compensation inequality on firm efficiency in the industry by constructing data envelopment analysis (DEA) and stochastic frontier analysis (SFA) scores to assess firm efficiency.

Because if its distinct benefits, the DEA method is frequently employed in the assessment of technological efficiency, total factor productivity, innovation efficiency, eco-efficiency, and resource usage rate. Numerous academics have enhanced DEA models in their research, in addition to the conventional CCR and BBC techniques. For example, the (Slack-Based Measure) SBM-DEA model considering slack variables [16,17], two-stage network data envelopment analysis (TSN-DEA) Model [18], Bootstrap-DEA model considering sampling method [19,20] and Bootstrap-DEA-Malmquist model [21,22], the Malmquist-Luenberger index considering non-expected outputs [23,24]. Chinese commercial banks’ efficiency was assessed by Duan et al. [25] using a two-stage DEA methodology. Using a three-stage DEA model, Suo and Zhang [26] investigated the technical efficiency of Chinese new energy listed businesses. Using a three-stage Bootstrap-DEA model, Dia et al. [27] evaluated the performance of Canada’s top 14 credit unions from 2007 to 2017 and examined the several elements that impacted that success. Lupu and Tiganasu [28] examined the effectiveness of the health system and the variables affecting it in the battle against neocoronitis in 31 European nations using the DEA and Tobit models. In order to compare the effectiveness of logistics systems, Lo Storto and Evangelista [29] employed the technique of DEA to measure the infrastructure efficiency values of EU countries for the years 2010–2017. Kang et al. [30] proposed hybrid two-stage network model and hybrid network DEA with shared input model to jointly measure the efficiency and effectiveness of metro transportation system.

When researching the elements that influence listed firms’ technological innovation efficiency, researchers primarily concentrate on both micro and macro dimensions. The labor structure, level of intellect, technical training, and equity structure are the primary areas of exploration for the micro viewpoint. The impact of ownership structure on the effectiveness of technological innovation in listed enterprises was examined by Zhang and Yan [31]. When Krišťáková et al. [32] examined the technological efficiency of wood-processing companies in Slovakia and Bulgaria, they discovered that both nations’ wood-processing industries are more economically efficient when they invest in cutting-edge technological advancements and step up their R&D efforts. In their analysis of the technological innovation efficiency of pharmaceutical companies, Lim and Min [33] discovered that an enterprise’s patent portfolio significantly positively affects its technological innovation efficiency. The effect of innovation capital and human resource investment on the technical innovation efficiency of businesses was examined by Wang et al. [34]. Zhao and Yang [35] looked at the relationship between intelligence level and the effectiveness of technological innovation as well as the associated mechanism analysis. Yin et al. [36] revealed the mechanism and heterogeneity of the impact of China’s bank-enterprise relationship on technological innovation efficiency by utilizing data on bank-enterprise relationship and technological innovation efficiency of 2008 firms over the period 2011–2020.

Macrolevel research mostly focuses on government subsidies, Internet development, corporate environments, and environmental regulations. In order to shed on the spatial heterogeneity and stage-specific characteristics of the impact of environmental regulation on technological innovation efficiency, Guan and Li [37] investigate the nonlinear effect of environmental regulation intensity on technological innovation efficiency at the R&D stage and the transformation stage. They also look at China’s two major regions, the east and the central and west. After conducting an empirical analysis of 3,204 Spanish enterprises between 2004 and 2005, Asimakopoulos et al. [38] came to the conclusion that the link between firms’ innovation efficiency and external sources of knowledge is structured like an inverted U. Based on the viewpoint of company financial asset allocation, Miao et al. [39] investigated the effects of government subsidies and business financial asset allocation on the efficiency of technological innovation. Im and Cho [40] assessed of the technological innovation efficiency of small and medium-sized enterprises(SMEs) in Korea’s manufacturing and service sectors revealed that SMEs’ technological innovation was positively impacted by the financial policy support provided by the government. In order to build a panel regression model of the efficiency of Internet development on technological innovation, Zhao et al. [41] used technology market development indicators as mediating variables. They also conducted an empirical analysis to determine the direct and indirect effects of Internet development on technological innovation in high-tech industries. The effect of digital finance technology development on the effectiveness of technical innovation in high-tech businesses was studied by Du and Wang [42]. The effect of business environment optimization on enterprise technological innovation was investigated by Li et al. [43]. Wan et al. [44] examined the main, moderating, mediating and nonlinear effects of government support and market competition on the innovation efficiency of China’s high-tech industries.

In terms of research objects, the main industries involved in the existing studies are: integrated circuit industry [45], Internet industry [46], innovative industry [47], equipment manufacturing industry [48], agriculture-related industries [49], strategic emerging industries [50], new energy enterprises [51] and so on. Using the microdata of 87 Chinese listed companies as an example, Lu and Meng [52] employ a three-stage DEA model to assess the technological innovation efficiency of the seven major sub-sectors and the strategic emerging industries as a whole. This allows them to determine the primary causes of the industry’s overall inefficiency in technological innovation as well as the variations in the technological innovation efficiency of the various sub-sectors. Su et al. [53] examined the convergence of the technological innovation efficiency of new energy firms by using the economic convergence theory to panel data from 2014 to 2018. They also measured the technological innovation efficiency of 78 new energy enterprises using the DEA-RAM model. Naeem et al. [54] used the DEA and Driscoll & Kraay techniques to analyze data for 19 African countries from 2000 to 2019 in order to study the effects of infrastructure, industrialization, and innovation on enhancing environmental efficiency in Africa.

In summary, the current body of literature on the measurement of enterprise technological innovation efficiency primarily uses data envelopment analysis (DEA) or stochastic frontier analysis (SFA) to examine the macro- and micro-level factors influencing enterprise technological innovation efficiency. The industries chosen for analysis are primarily focused on high-tech or strategically important emerging industries. When compared to previous research, this paper’s innovations mostly encompass the following areas: (1) To develop a solution for technological innovation and upgrading of the traditional manufacturing industry, traditional textile and apparel listed firms were chosen as the research object based on the perspective of technological innovation efficiency improvement. (2) A thorough analysis of the technological innovation efficiency of Chinese textile and garment listed firms is conducted from both static and dynamic perspectives, utilizing the three-stage DEA model and the Malaquist index model. (3) Using the Tobit panel regression model and impulse response function plot, this paper analyzes the influencing factors from the three aspects of macro factors, financial factors, and managerial factors for the technological innovation efficiency, excluding random noise interference and external environmental factors. At the same time, we pay attention to the differentiation caused by the internal heterogeneity of enterprises. In addition to offering some references to help the government create creative development plans, the aforementioned analysis aims to further enhance the research on the measurement and influencing elements of technical innovation efficiency in the textile and garment industry.

## 3 Measurement and evaluation of technological innovation efficiency

### 3.1 Research methodology

#### 3.1.1 Three-stage DEA modeling

(1) Phase I: DEA-BCC modeling

The DEA model is divided into input-oriented and output-oriented categories, and different orientations can be chosen according to the differences in analysis purposes. This paper uses the input-oriented DEA-BCC model to assess the technological innovation efficiency of textile and clothing enterprises because the input variables typically imply the strategy maker’s decisions, which are more actionable than the outputs, and the BCC model can measure the pure technical efficiency of each decision-making unit. It is constructed as follows:

$$\begin{array}{l} \min\left[\theta -\varepsilon ({\hat{e}}^{T}s^{-}+e^{T}s^{+})\right]\\ \left\{ \begin{array}{l} \sum_{j=1}^{n}x_{j}\lambda_{j}+s^{-}=\theta x_{0}\\ \sum_{j=1}^{n}y_{j}\lambda_{j}-s^{+}=y_{0}\\ \sum_{j=1}^{n}\lambda_{j}=1\\ \lambda_{j}\geq 0,j=1,2,\cdots ,n \end{array}\right. \end{array}$$ (1)

Where: *θ* is the efficiency value of the first *j* textile and garment enterprise $DMU_{j_{0}}$, *x*_*j*_ is the input variable, *y*_*j*_ is the output variable, *s*^+^ and *s*^−^ are the input and output slack variables, and *λ*_*j*_ is the weight coefficient of the input and output index values. Assuming that the optimal solution of BCC model is θ*, $\lambda_{j}^{*}$, s*^+^, s*^−^, then *e*^*T*^*s*^+^ represents the sum of inputs that need to be reduced for some inputs other than *θx*_0_; on the contrary, ${\hat{e}}^{T}s^{-}$ represents the sum of outputs that are insufficient for some outputs.

(2) Phase II: SFA modeling

For the purpose of regressing first-stage input excess *s*^−^ and external environmental parameters, the unique SFA regression model equation is:

$$\begin{array}{c} S_{ni}=f(Z_{i};\beta_{n})+\upsilon_{ni}+\mu_{ni}\\ \left(i=1,2,\cdots ,I;n=1,2,\cdots ,N\right) \end{array}$$ (2)

Where *S*_*ni*_ is the input slack of the *n* input of the *i* textile and clothing firm; *Z*_*i*_ is the environmental variable affecting the input; *β*_*n*_ is the coefficient of the environmental factor; *υ*_*ni*_ is the random disturbance term, $\upsilon_{ni}∼(0,\sigma_{ni}^{2})$; *μ*_*ni*_ is the residual term reflecting the degree of inefficiency in the production activities of the textile and clothing firm.

The input excess is modified in accordance with the equation, which is derived from the regression results:

$$\begin{array}{c} x_{ij}^{*}=x_{ij}+\left[\max(x_{i}{\hat{\beta }}^{n})-(x_{i}{\hat{\beta }}^{n})\right]+\left[\max({\hat{\upsilon }}_{ij})-{\hat{\upsilon }}_{ij}\right]\\ \left(i=1,2,\cdots ,m;j=1,2,\cdots ,n\right) \end{array}$$ (3)

Where $x_{ij}^{*}$ is the original input *x*_*ij*_ adjusted for the homogeneous environment. ${\hat{\upsilon }}_{ij}$ for the random error of the *DMU* on the input *n*, it is necessary to strip *υ*_*ij*_ from *υ*_*ij*_+*μ*_*ij*_, and according to the isolation method of Jondrow et al.[55], an estimate of *υ*_*ij*_ can be derived:

$$\begin{array}{c} E\left(\upsilon_{ij}|\upsilon_{ij}+\mu_{ij}\right)=S_{ij}^{-}-z_{i}\beta^{n}-E\left(\mu_{ij}|\upsilon_{ij}+\mu_{ij}\right)\\ \left(i=1,2,\cdots ,m;j=1,2,\cdots n\right) \end{array}$$ (4)

(3) Phase 3: Adapted DEA-BCC model.

The value of productivity of textile and garment enterprises, eliminating the impacts of noise and environmental factors, is produced by bringing the original data *y*_*ij*_ and the input data $x_{ij}^{*}$, modified by the second-stage SFA regression, back into the first-stage model for computation.

#### 3.1.2 Malmquist exponential modeling

In order to analyze changes in the technical efficiency of enterprise production, the Malmquist index analytical model, also known as the total factor productivity index analytical model [56], is a dynamic efficiency analysis method based on the DEA model. It can also decompose total factor productivity into three different categories: pure technical efficiency change index, scale efficiency index, and technical progress change index. The Malmquist index (M) model’s precise mathematical expression is displayed below:

$$M^{t+1}={\left[\frac{D^{t}(x^{t+1},y^{t+1})}{D^{t}(x^{t},y^{t})}•\frac{D^{t+1}(x^{t+1},y^{t+1})}{D^{t+1}(x^{t},y^{t})}\right]}^{1/2}$$ (5)

Where (*x*,*y*) denotes the input and output variables in each period; *D*^*t*^(*x*^*t*+1^,*y*^*t*+1^) and *D*^*t*^(*x*^*t*^,*y*^*t*^) denote the distance functions of inputs *x* and outputs *y* at *t*+1 and *t* respectively, using the technology of *t* as a reference; *D*^*t*+1^(*x*^*t*+1^,*y*^*t*+1^) and *D*^*t*+1^(*x*^*t*^,*y*^*t*^) denote the distance functions of inputs *x* and outputs *y* at *t*+1 and *t* respectively, using the technology of *t*+1 as a reference, and the following equations have the same meanings. If the measured value is less than 1, it means that the resource utilization efficiency decreases from the period of *t* to the period of *t*+1; if the measured value is greater than 1, it means that the resource utilization efficiency increases from the period of *t* to the period of *t*+1.

The Malmquist index can be further broken down into the index of change in technical efficiency (EFFCH) and the index of change in technical advancement (TECHCH), assuming constant returns to scale. Eq (5) can be written as follows.


$$\begin{array}{l} M^{t+1}=EFFCH\times TRCHCH\\ \;=\frac{D^{t+1}(x^{t+1},y^{t+1})}{D^{t}(x^{t},y^{t})}•{\left[\frac{D^{t}(x^{t},y^{t})}{D^{t+1}(x^{t},y^{t})}•\frac{D^{t}(x^{t+1},y^{t+1})}{D^{t+1}(x^{t+1},y^{t+1})}\right]}^{1/2} \end{array}$$ (6)

If variable returns to scale are considered, the index of technical efficiency change (EFFCH) can be further decomposed into the index of pure technical efficiency change (PECH) and the index of scale efficiency (SECH):

$$\begin{array}{l} M^{t+1}=EFFCH\times TECHCH\\ \;=PECH\times SECH\times TECHCH\\ \;=\frac{D_{r}^{t+1}(x^{t+1},y^{t+1})}{D_{r}^{t}(x^{t},y^{t})}•\left[\frac{D_{r}^{t}(x^{t},y^{t})}{D^{t}(x^{t},y^{t})}/\frac{D_{r}^{t+1}(x^{t+1},y^{t+1})}{D^{t+1}(x^{t+1},y^{t+1})}\right]\\ \;•{\left[\frac{D^{t}(x^{t},y^{t})}{D^{t+1}(x^{t},y^{t})}•\frac{D^{t}(x^{t+1},y^{t+1})}{D^{t+1}(x^{t+1},y^{t+1})}\right]}^{1/2} \end{array}$$ (7)

Where *r* denotes variable returns to scale, $D_{r}^{t+1}(x^{t+1},y^{t+1})$ denotes the distance function between inputs *x* and outputs *y* at *t*+1 in the case of variable returns to scale, using the technology at *t*+1 as a reference; $D_{r}^{t}(x^{t},y^{t})$ denotes the distance function between inputs *x* and outputs *y* in the case of variable returns to scale, using the technology at *t* as a reference; and the rest of the variables have the same meanings as those in Eq (5).

### 3.2 Selection of indicators

This study is based on the technological innovation efficiency at the micro-enterprise level. Building upon the research by Guo et al. [57], it categorizes input factors into productive inputs, operational inputs, and research and development inputs. Similarly, outputs are divided into profit-oriented outputs and technical outputs.

(1) Input indicators

Productive inputs comprise investments in fixed assets and employee contributions, with capital and labor being the foundational elements of corporate production and operational activities. The quality of a company’s assets and workforce significantly impacts the efficiency of technological innovation. Following the methodology of Meng and Xu [58], this is represented by the fixed assets and total number of employees as reported in the annual reports of listed companies.

Operating inputs refer to the various forms of investment activities undertaken by companies to achieve production and operational goals. Such inputs aim to generate value through effective business operations, exceeding the costs required for these operations, thereby generating profits for the company. This paper uses operating costs and accounts receivable from the annual reports of listed companies to represent the operating inputs of a business [59].

Research and development (R&D) inputs represent the investment of funds, resources, and effort by companies in the R&D sector, aimed at enhancing market competitiveness and sustainable development capabilities through innovation and technological improvements. This paper follows the approach of Han et al. [60], using the total R&D expenditures as reported in the annual reports of listed companies to denote R&D spending.

(2) Output indicators

Profitable output refers to the economic benefits gained by a company through its operational activities, typically manifested as growth in profits and an increase in profit margins. The core of profitable output lies in how to effectively utilize resources, optimize business strategies and management methods, to achieve cost control and revenue maximization. This paper follows the research method of Liu et al. [59], using net profit and operating income as reported in the annual reports of listed companies to represent this.

Technical output refers to various forms of results generated through scientific and technological activities, including direct outputs such as scientific papers and patent production, as well as indirect outputs such as the economic and social benefits of scientific and technological achievements. This paper uses the total intangible assets as reported in the annual reports of listed companies to represent this [61].

(3) Environment indicators

The ways in which various environmental factors influence the technological innovation efficiency of textile and clothing listed enterprises differ, and these factors must meet the requirements of the "separation hypothesis" [62,63]. This means that while these variables affect the enterprise’s daily production activities, they are outside its control and primarily affect the input and output factors of the listed enterprises, which in turn affects their production efficiency indirectly.

To effectively analyze the development environment and production characteristics of the textile and apparel industry, this paper selects environmental influencing factors from both internal and external aspects. After referencing the research of scholars [58,61,64], internal environmental factors are represented by the shareholding ratio of the largest shareholder of listed companies and the establishment years of the company. External environmental factors are represented by the economic development level of the region where the company is located and the degree of foreign trade dependence. Among them, the regional economic development level is represented by the per capita GDP of the region where the company is located, and the degree of foreign trade dependence is represented by the percentage of the total import and export volume of the region where the company is located to the total GDP of the region.

The specific descriptions of each indicator are shown in Table 1.

**Table 1:** Technological innovation efficiency evaluation indicator system.

Variable type		Variable name (Unit)	Calculation method
**Inputs**	Productive	Fixed assets (CNY)	Total fixed assets in annual reports of listed companies
		Total number of employees (Persons)	Total number of employees in annual reports of listed companies
	Operational	Operating costs (CNY)	Operating costs in annual reports of listed companies
		Accounts receivable (CNY)	Accounts receivable in annual reports of listed companies
	R&D	R&D expenditure (CNY)	Total R&D expenditure in annual reports of listed companies
**Outputs**	Profitable	Net profit (CNY)	Net profit in annual reports of listed companies
		Operating income (CNY)	Operating income in annual reports of listed companies
	Technical	Intangible assets (CNY)	Total intangible assets in annual reports of listed companies
**Environment variable**	Internal environment factors	Degree of shareholding concentration (%)	Proportion of shares held by the largest shareholder in the annual reports of listed companies
		Years of enterprise establishment (Years)	(Current year—year of establishment) +1
	External environment factors	Level of economic development (CNY)	GDP per capita in the region where the enterprise is located
		Degree of foreign trade dependence (%)	Percentage of total exports and imports of the region in which the enterprise is located in relation to the total GDP of the region

### 3.3 Data sources and processing

This paper selects listed textile and garment enterprises as the research object, mainly based on two considerations. On the one hand, listed companies have higher economic benefits and added value, and are mostly the leaders of the relevant industries with stronger growth, which is highly representative and persuasive. On the other hand, the relevant data disclosed by listed companies every year have been examined by accounting firms and relevant departments of the Securities and Futures Commission, and the data are more credible.

According to the "Guidelines on Industry Classification of Listed Companies" (revised in 2012) issued by the Securities and Futures Commission (SFC), the enterprises with the first two codes of 17 and 18, i.e., enterprises listed in China’s A-share market under the two major categories of "textile" and "textile and garment, apparel", totaled 93 companies. Excluding 26 enterprises listed for less than 5 years, 3 enterprises that had been *ST or ST during the inspection period and 4 enterprises whose relevant data had not been disclosed, there were a total of 60 enterprises in the sample that met the requirements, of which 31 were in the textile industry and 29 were in the apparel industry. The 60 sample enterprises screened are mainly concentrated in the eastern coastal region, of which 21 are from Zhejiang Province (35%), 7 each from Shanghai, Jiangsu Province and Guangdong Province (11.67% each), 4 each from Fujian Province and Shandong Province (6.67% each), 3 each from Beijing City and Anhui Province (5% each), 2 each from Hunan Province (3.33% each), 1 each from Henan Province and 1 each in Sichuan Province (1.67% each). The Choice database provides information on input and output indicators, while the National Bureau of Statistics, province statistical bureau websites, and annual reports of publicly traded enterprises provide information on associated environmental factors.

There are four basic approaches to handling the negative values that occur in practice because the DEA model necessitates that the pertinent indicator data be all non-negative: (1) the data to the (0, 1) interval using dimensionless mapping [65]; (2) converting the negative value into a small enough positive integer [66]; (3) primary row transformation, meaning that negative values are removed by organizing the output and input metrics into matrices and applying an elementary row transformation to the matrices [67]; (4) data shifting, meaning that all data is "added values" in order to change negative values to positive values [68]. By comparing the advantages and disadvantages of each method, this paper finally chooses to dimensionless the data to eliminate the effect of negative values without changing the original data, and use interpolation to make up the missing individual data.

### 3.4 Three-stage DEA efficiency measurement results

#### 3.4.1 Statistical description of input-output indicators

This study uses samples of 60 listed textile and apparel firms covering the period from 2013 to 2022. The statistical description of each indicator is performed on the panel data that has been composed, and the results are displayed in Table 2.

**Table 2:** Descriptive statistics of technical innovation efficiency evaluation indicators.

Variable name (Unit)	Obs	Std. dev.	Mean	Min	Max
Fixed assets (CNY)	660	131806.2	105215.2	238.82	857526.2
Total number of employees(Persons)	660	6102.57	5635.09	75	41167
Operating costs (CNY)	660	342001.6	238786.1	11681.25	2522035
Accounts receivable (CNY)	660	43154.76	35947.06	786.86	375425.6
R&D expenditure (CNY)	660	6727.32	6635.17	176.75	41365.36
Net profit (CNY)	660	66031.17	27267.87	-141347.5	720657.9
Operating income (CNY)	660	427515.5	344263	20344.11	2715534
Intangible assets (CNY)	660	46168.94	22565.19	56.38	454932.8
Degree of shareholding concentration (%)	660	17.18	36.97	7.8	97.28
Years of enterprise establishment (Years)	660	6.33	18.22	3	41
Level of economic development (CNY)	660	3.35	9.01	2.96	20.38
Degree of foreign trade dependence (%)	660	27.54	56.06	5.79	135.43

The "homogeneity" concept must be met by the model’s indicators in order for the DEA to function [69]. As a result, the Pearson correlation test is run for each indicator, and Table 3 displays the results. As can be shown, all of the chosen indicators exhibit a strong positive correlation at the 1% level. This suggests that the indicators used in this study adhere to the "homogeneity" principle and can be used to further build the DEA model.

**Table 3:** Input-output correlation tests.

	Fixed assets	Total number of employees	Operating costs	Accounts receivable	R&D expenditure
**Net profit**	0.54^***^	0.423^***^	0.477^***^	0.533^***^	0.668^***^
**Operating income**	0.799^***^	0.825^***^	0.976^***^	0.945^***^	0.832^***^
**Intangible asset**	0.562^***^	0.689^***^	0.729^***^	0.777^***^	0.645^***^

Note

***, **, and * represent 1%, 5%, and 10% significance levels, respectively.

#### 3.4.2 Phase I: DEA-BCC model results

The industry average was determined by year, and the efficiency values of the first stage of the DEA-BCC model are displayed in Table 4. The comprehensive efficiency (TE), pure technical efficiency (PTE), and scale efficiency (SE) of 60 listed textile and garment enterprises were calculated based on the DEA-BCC model with the aid of DEAP2.1 software.

**Table 4:** Average value of technological innovation efficiency over the years.

Year	TE	PTE	SE
**2013**	0.924	0.957	0.966
**2014**	0.931	0.947	0.982
**2015**	0.938	0.948	0.989
**2016**	0.936	0.951	0.983
**2017**	0.883	0.934	0.945
**2018**	0.866	0.937	0.925
**2019**	0.897	0.919	0.974
**2020**	0.881	0.909	0.968
**2021**	0.868	0.899	0.964
**2022**	0.887	0.911	0.972
**Mean**	0.901	0.931	0.967

Table 4 shows that, for the period of 2013–2022, the average comprehensive efficiency value of listed textile and apparel enterprises ranges from 0.866 to 0.938. The lowest value of 0.866 was recorded in 2018, the highest value of 0.938 was recorded in 2015, and the average value overall is 0.901. The average scale efficiency value fluctuates between 0.925 and 0.989, with an overall mean value of 0.967; the overall efficiency value is higher than the average pure technical efficiency value. The average pure technical efficiency value has a variation range of 0.899 to 0.957, and the overall average value is 0.931.

#### 3.4.3 Phase II: SFA regression results

The degree of equity concentration, the number of years of establishment, the level of economic development, and the degree of dependence on foreign trade are taken as the explanatory variables to establish a similar SFA model. This model is calculated by using the Frontier4.1 software to study the influence of environmental variables on the input slack variables. The slack variables of fixed assets, total employees, operating costs, accounts receivable, and R&D expenditures are taken as the explained variables.

Table 5 shows that there is a significant difference in the management efficiency of textile and clothing listed enterprises, warranting the SFA regression. The values of *σ*^2^ and *γ* pass the significance test, and the values of *γ* are greater than 0.6, indicating that management inefficiency does exist and accounts for a large proportion. Overall, the LR test for redundancy of each input variable passed the significance test at the 1% level, suggesting that statistical noise and environmental variables need to be eliminated in order to fully understand the significant impact that each environmental variable has on the technological innovation efficiency of textile and apparel firms.

**Table 5:** SFA model environmental variables regression results.

Name	Fixed assets	Total number of employees	Operating costs	Accounts receivable	R&D expenditures
**Constant term**	-278.35*** (-9.75)	-1146.27* (-1.89)	-22495.12*** (-498.84)	-4317.13*** (-153.12)	-1746.15** (-2.01)
**Degree of shareholding concentration**	-443.33*** (-3.63)	-7.85 (-1.26)	-249.13* (-1.68)	-87.45** (-2.07)	-16.04** (-2.27)
**Years of enterprise establishment**	1689.81*** (22.59)	56.11** (2.37)	1856.62*** (6.33)	283.34** (1.98)	101.94*** (2.63)
**Level of economic development**	-2010.19*** (-6.51)	-34.26 (-1.02)	-18.33 (0.14)	429.11 (1.52)	-72.61 (-1.30)
**Degree of foreign trade dependence**	-145* (-1.65)	2.25 (0.52)	-235.23** (-2.46)	-67.23** (-2.14)	1.32 (0.19)
***σ* ^2^**	6494895200*** (6494894900)	12604696*** (12508868)	8181444500*** (8181444200)	473605350*** (473604430)	29102137*** (28461718)
***γ***	0.82*** (81.09)	0.86*** (108.58)	0.76*** (71.13)	0.62*** (29.03)	0.84*** (115.15)
**Log**	-7896.83	-5761.91	-8064.34	-7256.32	-6073.18
**LR**	504.36***	625.78***	380.54***	220***	564.57***

Note: ***, **, and * indicate significant at the 1%, 5%, and 10% levels, respectively, and values in parentheses are the corresponding t-values. All of the above models passed the 1% LR one-sided likelihood ratio test.

#### 3.4.4 Phase III: Adjusted DEA-BCC results

**Fig 2:**
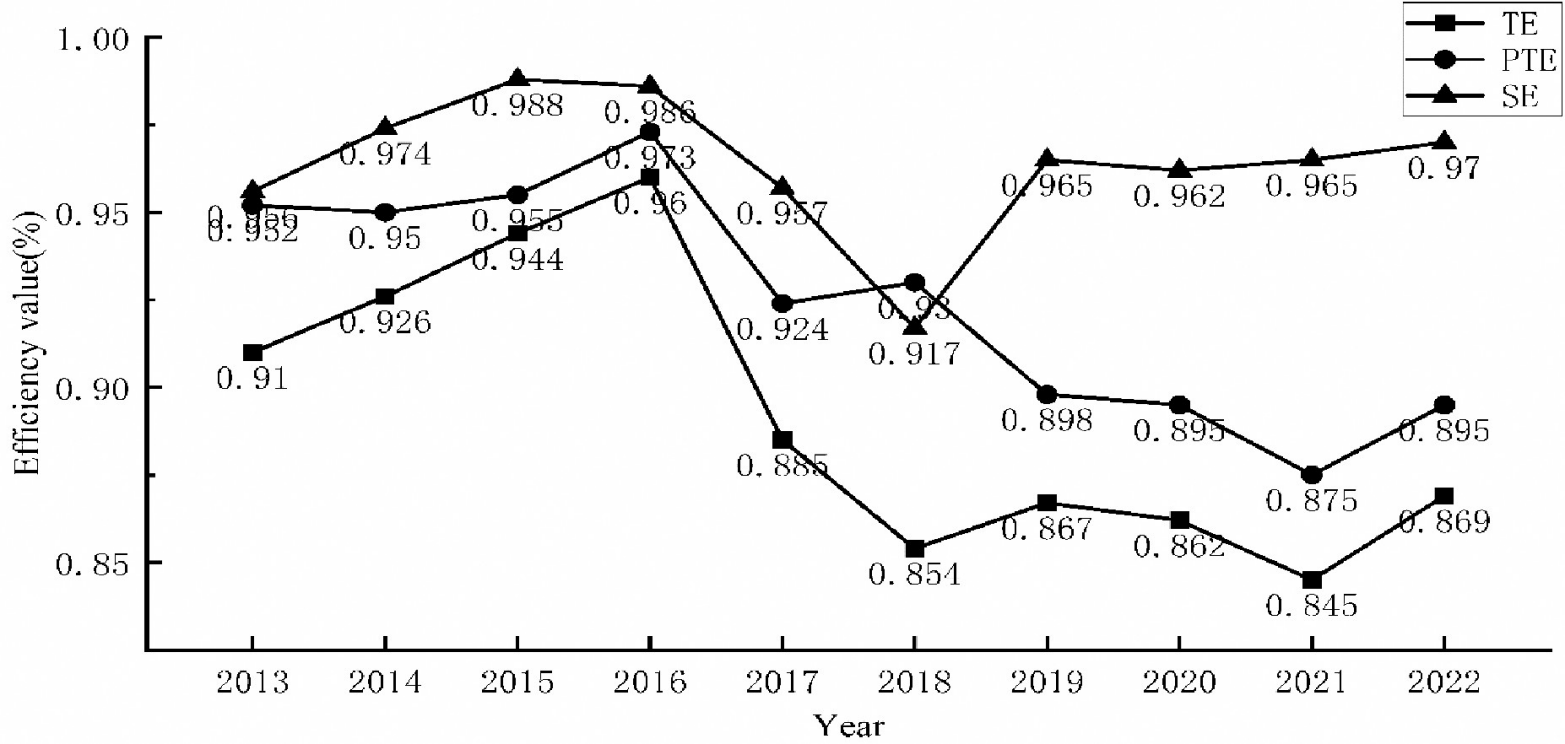
Adjusted average annual technological innovation efficiency.

The DEA-BCC model was used to recalculate the comprehensive efficiency (TE), pure technical efficiency (PTE), and scale efficiency (SE) of listed textile and garment enterprises, excluding environmental factors, using the data of adjusted input variables and original output variables from the second stage. The mean values of the results are displayed in Fig 2.

After removing outside disruptive variables, the results of an enterprise’s technical innovation efficiency operation can more precisely reflect the state of technology at that company. Fig 1 shows that, when compared to the first stage, the adjusted average comprehensive efficiency, average pure technical efficiency, and average scale efficiency of listed textile and garment enterprises have all decreased. This suggests that the external environment has an impact on textile and garment enterprises, and that the technological innovation efficiency of enterprises has been artificially increased. The average total efficiency of listed textile and apparel firms has fluctuated between growth and decline, growth and decline, growth and decline, between 2013 and 2022. The overall trend is dropping, with fluctuations in the decline trend and an average comprehensive efficiency value ranging from 0.845 to 0.96. After reaching a peak in 2016, the average integrated efficiency value started to drastically decrease, mostly as a result of the country’s supply-side structural adjustment and the trade spat between the United States and China. After a brief recovery in 2018, it hit rock bottom once more as a result of the New Crown Epidemic.

The average comprehensive efficiency and pure technical efficiency have a similar trend. Over the last ten years, the average pure technical efficiency has fluctuated between 0.875 and 0.973. The overall value is lower than the average scale efficiency, which has limited the potential for listed textile and apparel enterprises’ average comprehensive efficiency value to increase. In addition to the two years of decline in 2017 and 2018, the overall value of the average scale efficiency is relatively high, reaching an average of 0.964. This is in comparison to the average pure technical efficiency value, which is higher, and it helps to enhance the average comprehensive efficiency value of the enterprise. It also confirms that management-driven technological innovation in China’s textile and garment enterprises is inefficient and cannot be sustained.

**Fig 3:**
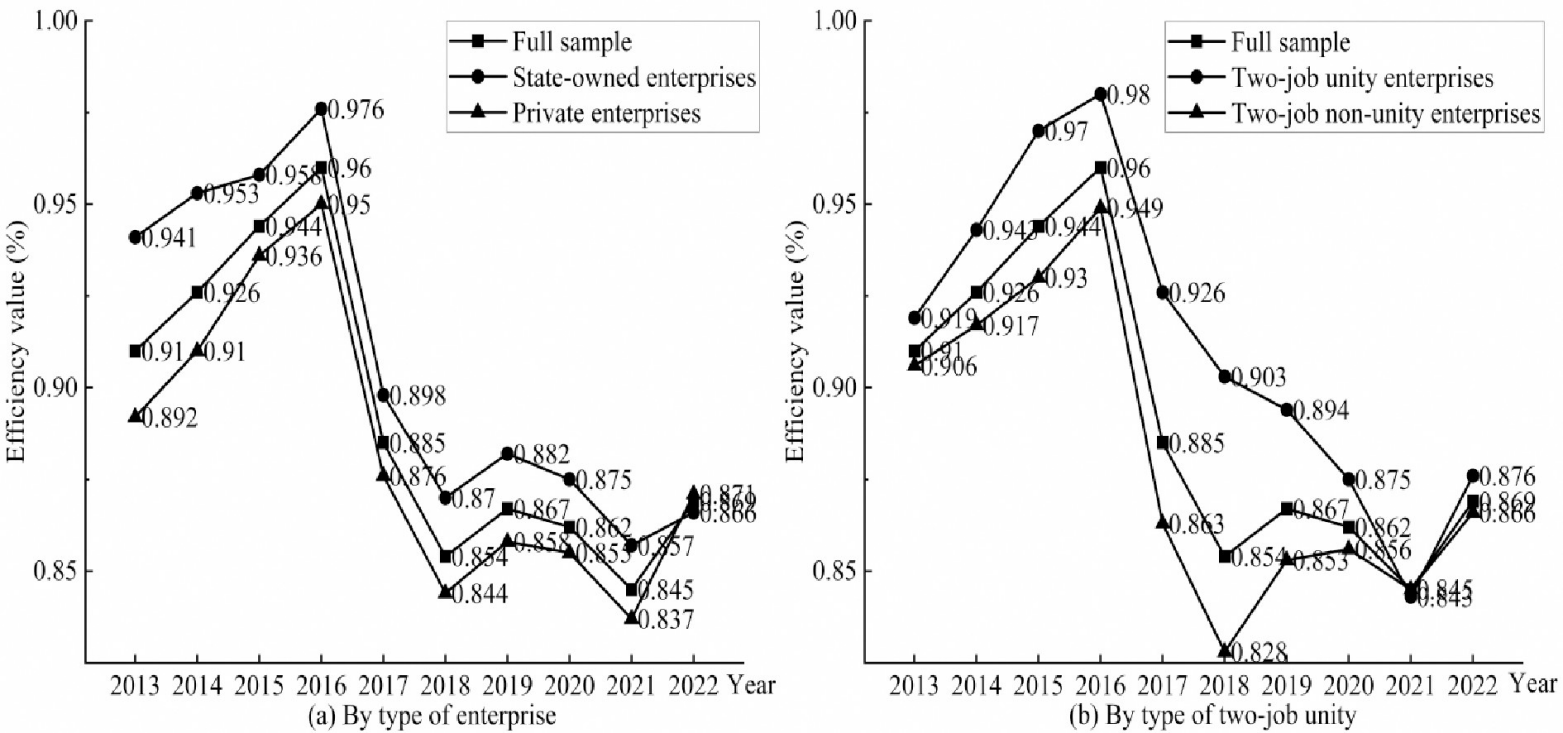
Classification by enterprise heterogeneity.

The enterprise can be classified as either state-owned or private based on whether it has state-owned components. Additionally, it can be further classified as two-job unity enterprises or two-job non-unity enterprises based on whether the person holding the positions of chairman and general manager is the same. After accounting for environmental considerations, the average comprehensive efficiency value of listed textile and garment firms is grouped by enterprise type and two-job indication to create a line graph, as illustrated in Fig 3.

The average comprehensive efficiency value of state-owned and private businesses has fluctuated over the past ten years in a way that has been consistent with the overall average efficiency value. Both have shown a downward trend of fluctuation, with growth being followed by decline, then growth, and finally decline, with the fluctuation amplitude of the three as a whole remaining consistent. Overall, state-owned businesses have higher average comprehensive efficiency values than private businesses, with the biggest difference observed in 2013. However, as time went on, this difference gradually closed, and by 2022, the two essentially overlapped. This shows that listed textile and apparel companies with state-owned components remain at the forefront of the market and pull innovation in the industry as a whole. However, in recent years, private companies have also started to concentrate on increasing the efficiency of technological innovation.

For the two-job classification of listed textile and apparel companies, the trend of change in the value of comprehensive efficiency for both two-job unity and two-job non-unity companies is consistent with the trend of change in the value of overall average comprehensive efficiency from 2013 to 2022, but the overall change is more soothing for two-job unity companies. Overall, the average comprehensive efficiency value of enterprises with two-job unity is higher than that of enterprises two-job non-unity, which indicates that two positions for the same person is conducive to enhancing the technological innovation efficiency of listed textile and apparel enterprises, but this advantage begins to fade away over time, especially in 2021, when the average comprehensive efficiency values of the two are basically overlapping.

#### 3.4.5 Horizontal comparison analysis among industries

To gain a better understanding of the textile and apparel industry’s position within the industry, this paper also computes the technological innovation efficiency of the manufacturing industry and its various subsectors. These figures are used as a control group to compare the textile and apparel industry side by side. A total of 1,833 businesses in the manufacturing sector meet the requirements of the sample, and 15 subsectors are obtained based on the number of samples of the various subsectors and similarities between the deletion and merger. The sample data of businesses in the manufacturing sector and various subsectors is also selected in accordance with the Guidelines for the Industrial Classification of Listed Companies (2012 Revision) issued by the Securities and Futures Commission. This excludes businesses that have been listed for less than five years, have been *ST or ST during the inspection period, and have not disclosed relevant data. The Choice database, annual reports of publicly traded firms, the National Bureau of Statistics, and the websites of regional statistical offices are the sources of the data for the input and output indicators.

**Fig 4:**
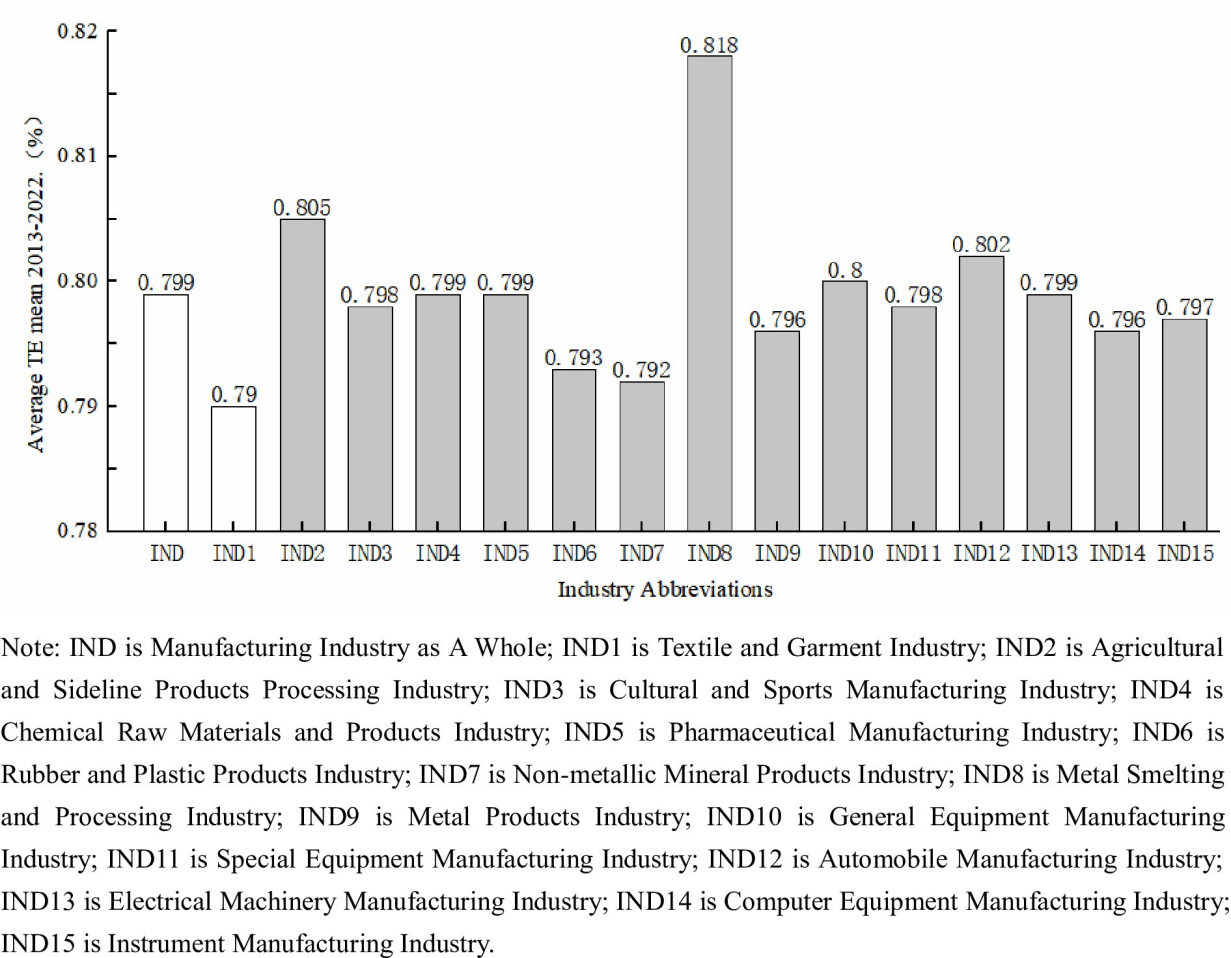
Average combined efficiency mean for manufacturing and subsectors, 2013–2022.

Fig 4 presents a comparison of the average comprehensive efficiency mean value for the manufacturing industry and its various subsectors from 2013 to 2022. The manufacturing industry as a whole has an average comprehensive efficiency mean value of 0.799 over the past ten years, and among the various subsectors, all but the textile and garment enterprises have average comprehensive efficiency mean values higher than 0.79, with the lowest average value being the non-metallic mineral products industry at 0.792 and the highest being the metal smelting and processing industry at 0.818. As such, the textile and garment industry’s overall technological innovation efficiency value is relatively low when compared to the manufacturing industry as a whole and its various sub-sectors. This significantly reduces the manufacturing industry’s overall technological innovation efficiency value.

### 3.5 Malmquist index model analysis

The three-stage DEA model measures the static relative efficiency of different decision-making units in the same period, while the Malmquist index model analyzes the dynamic efficiency of the data of each decision-making unit in different periods [70]. Thus, this paper introduces the Malmquist index model to further explore the total factor productivity change and the corresponding technological progress and technological efficiency change from a dynamic perspective. It also discusses these topics from the perspectives of time-series change and enterprise heterogeneity, respectively. This is based on the static analysis of technological innovation efficiency of textile and apparel listed enterprises by the three-stage DEA model. The Malmquist index model is calculated for each input and output variable using the software DEAP2.1.

#### 3.5.1 Analysis from the point of view of time series changes

Table 6 displays the total factor productivity index for textile and apparel businesses as well as the breakdown of that index from 2013 to 2022. The total factor productivity of textile and apparel firms has declined by 1.7% based on the overall mean value, indicating a poor degree of development overall. The breakdown reveals that, at 0.9% and 0.8%, respectively, the average values of technological efficiency and advancement are declining, making it impossible for these variables to effectively support the expansion of total factor productivity in listed textile and apparel companies. The rate of decline for both pure technical efficiency and scale efficiency, which further decompose technical efficiency, is 0.4%, meaning that there is little chance of promoting technical efficiency.

**Table 6:** Malmquist index and its decomposition by year.

Year	Total factor productivity variation index	Technological progress variation index	Technical efficiency variation index	Pure technical efficiency variation index	Scale efficiency variation index
**2013–2014**	0.981	0.977	1.003	1.004	1
**2014–2015**	1.073	1.072	1	1.003	0.997
**2015–2016**	0.881	0.873	1.01	1.018	0.993
**2016–2017**	0.982	1.03	0.953	0.954	0.999
**2017–2018**	1.016	1.02	0.996	1.009	0.988
**2018–2019**	0.861	0.89	0.968	0.976	0.991
**2019–2020**	1.015	1.033	0.982	1.006	0.977
**2020–2021**	0.997	0.996	1.002	0.988	1.014
**2021–2022**	1.059	1.045	1.014	1.005	1.009
**Mean**	0.983	0.991	0.992	0.996	0.996

The total factor productivity change index for textile and apparel companies from 2013 to 2022, expressed in terms of years, has a "W" fluctuation state. The years that show a rising trend are 2014–2015, 2017–2018, 2019–2020, and 2021–2022. However, growth rates are generally not very high, with 2014–2015 showing a relatively high growth rate of only 7.3%. The breakdown of total factor productivity in the growth years indicates that the primary source of growth power is the advancement of technological progress. When it comes to the years that total factor productivity is dropping, the decline rate in 2015–2016 is the highest, coming in at 11.9%. A closer look at the declining years also shows that the decline in technical advancement is the primary cause of the decline. It is evident that the total factor productivity of listed textile and apparel firms rises and falls mostly due to changes in the index of technological advancement, and enterprises should continue to strengthen their capacity to advance technologically.

#### 3.5.2 Analysis from the perspective of firm heterogeneity

Table 7 displays the findings of grouping textile and garment listed firms’ total factor productivity index and its decomposition based on two sets of indicators: enterprise type and two-job. Both state-owned and private businesses’ total factor productivity is declining; state-owned businesses’ decline was 1.9%, while private businesses’ decline was 1.6%. Upon closer examination, it becomes evident that the decline in total factor productivity of state-owned enterprises is primarily caused by a decline in technological efficiency, whereas the decline in technological progress is the primary cause of the decline in total factor productivity of private enterprises. The total factor productivity of businesses with two-job unity was down 2.1% in comparison to the 1.5% decline in businesses with two-job non-unity. Further analysis shows that the reasons for both declines were the same, primarily related to the slowdown in technological advancement.

**Table 7:** Malmquist index of firm heterogeneity and its decomposition.

Categorization	Total factor productivity variation index	Technological progress variation index	Technical efficiency variation index	Pure technical efficiency variation index	Scale efficiency variation index
**State-owned enterprises**	0.981	0.991	0.990	0.994	0.996
**Private enterprises**	0.984	0.990	0.993	0.996	0.997
**Two-job unity enterprises**	0.979	0.988	0.991	0.996	0.995
**Two-job non-unity enterprises**	0.985	0.992	0.993	0.995	0.997

## 4 Analysis of factors influencing the efficiency of technological innovation

### 4.1 Research hypotheses

The role that internal management plays in an organization’s development process is significant since it directly influences the degree of development of the organization and establishes the internal production efficiency. Enterprise internal management is not limited to improving the cohesion within the organization; it also plays a significant role in improving the efficiency of day-to-day operations and achieving efficient resource allocation, particularly for textile and apparel companies listed on the resource endowment of enterprises. This is necessary for the enterprises to develop quickly. The efficiency of technological innovation is also greatly influenced by the internal management of businesses. Effective internal management can effectively allocate financial, material, and human resources to technology research and development, improve the effectiveness of this process, and ultimately increase the efficiency of technological innovation. As a result, this paper proposes H1 hypothesis:

H1: The higher the level of internal management of the firm the more favorable it is to improve the efficiency of technological innovation.

The development of enterprises, particularly the financial management of listed firms, is significantly influenced by financial considerations. This is also the case for listed textile and clothing industries. Better financial performance of the business indicates greater profitability and development potential. It also means that the business can invest more in relevant technology research and development, giving it more financial resources to overcome high precision technology and boost the effectiveness of its technological innovation. Furthermore, the enterprise’s solvency and operating capacity are also reflected in its financial management. These factors not only help to prevent debt repayment crises for the business, but also ensure that its technological innovation will continue to grow as a result of uninterrupted research and development. Therefore, this paper proposes H2 hypothesis:

H2: A higher level of corporate financial management is more conducive to improving the efficiency of technological innovation.

### 4.2 Modeling

#### 4.2.1 Tobit model

The Tobit model is a type of regression model that is restricted to dependent variables, meaning that its values can only be taken if the dependent variable satisfies specific requirements. The DEA model yielded technological innovation efficiency values for textile and apparel enterprises that fall between 0 and 1. This is because the explanatory variables are drawn from the truncated discrete distribution data. If the Ordinary Least Squares (OLS) method is employed for the direct regression of the model, there is a possibility of bias and inconsistent parameter estimation [71]. As a result, the maximum likelihood estimation (ML) approach is used in this work to regress the Tobit model in the article, so avoiding the bias and inconsistent parameter estimation issues. We built a multivariate linear regression model based on Tobit by using the value of technological innovation efficiency of listed textile and apparel enterprises as measured by the three-stage DEA model as the explained variable and the factors affecting the changes in the value of technological innovation efficiency as the explanatory variables. This is how it is expressed:

$$Y_{i}=\beta_{0}+\beta^{T}X_{i}+\varepsilon_{i}$$ (8)

Where *i* = 1,2,3,⋯, represents different decision units, *Y*_*i*_ is the restricted dependent variable for the *i* observations of the sample data, *X*_*i*_ is the explanatory variable, *β*_0_ is the constant term, *β*^*T*^ is the vector of unknown parameters, and *ε*_*i*_ is the residual term and satisfies *ε*_*i*_~*N*(0,*σ*^2^).

#### 4.2.2 Selection of variables

This paper uses the three-stage DEA model’s derived comprehensive efficiency (TE), pure technical efficiency (PTE), and scale efficiency (SE) as the explained variables. This allows the explanation to more accurately reflect which explanatory variables are affected as well as real changes in the technological innovation efficiency of textile and apparel listed enterprises.

As the basic unit of the textile and garment industry, the technological innovation efficiency of listed textile and garment enterprises will be affected by various aspects. On the basis of the three-stage DEA model to eliminate the influence of external environmental factors, the Tobit model will focus on the influence of internal factors on the technological innovation efficiency of listed textile and garment enterprises, which mainly includes management factors and financial factors. The management factors are chosen to represent the caliber of workers, the enterprise’s age, management expenses, and equity concentration, while the financial factors are chosen to represent profitability, solvency, development potential, and operating capacity. Additionally, the enterprise type and the type of two-job unity are chosen as grouping variables. Table 8 provides a detailed description of each indicator.

**Table 8:** Tobit model indicator system.

Variable type		Variable name (Unit)	Variable symbol	Calculation method
**Explained variables**		Comprehensive efficiency	Te	Calculated by the three-stage DEA model
		Pure technical efficiency	Pte	Calculated by the three-stage DEA model
		Scale efficiency	Se	Calculated by the three-stage DEA model
**Explanatory variable**	**Management factors**	Employee quality (%)	Huma	Percentage of personnel at the tertiary level and above
		Age of business (%)	Age	Logarithmic number of years of business establishment
		Management costs (%)	Admin	Corporate overhead rate
		Shareholding concentration (%)	Cr5	Shareholding ratio of top five shareholders
	**Financial factors**	Profitability (%)	Prof	Principal components are extracted from net profit margin, cost-expense margin, operating profit margin, and total return on equity
		Development capacity (%)	Deve	Principal components are extracted from the year-on-year growth rate of operating revenue, year-on-year growth rate of net profit, year-on-year growth rate of return on net assets, and year-on-year growth rate of total profit
		Solvency (%)	Debt	Principal components are extracted from cash ratio, equity multiplier, year-on-year growth rate of operating income, quick ratio, current ratio
		Operating capacity (%)	Oper	Principal components are extracted from current asset turnover, return on net assets, current ratio, total asset turnover ratio
**Grouping variable**		Type of enterprises	Type	1 for state-owned enterprises, 0 for private enterprises
		Type of two-job unity	Ceo	1 if the chairman and general manager are the same, 0 if they are not

Since there are numerous indicators that can capture the financial aspects of profitability, development ability, solvency, and operating ability, using just one indicator will not be able to accurately and completely reflect the true state of the business, while using multiple indicators will likely result in multicollinearity. Principal component analysis is used to identify the major components from a large number of variables to indicate the ability of these four characteristics. This allows the impact of these four aspects on the technological innovation efficiency of listed textile and apparel firms to be objectively reflected. Net profit margin, cost and expense margin, operating profit margin, and total return on assets are the criteria for profitability; year-over-year growth rates of operating income, net profit, growth rate of return on net assets, and year-over-year growth rate of total profit are the criteria for development capability; cash ratio, equity multiplier, year-over-year growth rate of operating income, quick ratio, and current ratio are the criteria for solvency; and year-over-year growth rates of operating income, return on net assets, current ratio, and total asset turnover are the criteria for operational capability. The data were analyzed using principal component analysis using SPSSPRO software. The number of principle components was calculated using the eigenvalue and cumulative variance contribution rate, and the findings are displayed in Table 9. As can be seen from the principal component analysis results, the chosen indicators are amenable to principal component analysis; however, due to space constraints, the precise computation procedure will not be reproduced in this study.

**Table 9:** Results of principal component analysis.

Composite indicators	Number of principal components	KMO value	Bartlet’s test	Eigenvalue	Cumulative variance contribution
Profitability	1	0.787	0.000***	3.626	90.65%
Development capacity	2	0.636	0.000***	3.08 and 0.898	99.94%
Solvency	2	0.705	0.000***	3.384 and 0.93	86.28%
Operating ability	2	0.71	0.000***	2.812 and 0.744	88.91%

Note: ***, **, and * represent 1%, 5%, and 10% significance levels, respectively.

The above article’s 60 listed textile and apparel businesses are still used as samples for the Tobit model analysis. These businesses are arranged into panels based on data from 2013 to 2022. The explanatory variables’ data originates from the three-stage DEA model’s calculation results, while the grouping variables and explanatory variables’ data are sourced from the Choice database, the annual reports of the listed businesses, the websites of the National Bureau of Statistics, and the statistical bureaus of each province. Table 10 displays the statistical explanations of each indicator. To prevent the impact of extreme values, 1% tailing was applied to the continuous data.

**Table 10:** Descriptive statistics of tobit indicators.

Variable name (unit)	Obs	Std. Dev.	Mean	Min	Max
Comprehensive efficiency	600	0.893	0.104	0.639	1
Pure technical efficiency	600	0.925	0.088	0.674	1
Scale efficiency	600	0.965	0.046	0.785	1
Employee quality (%)	600	29.75	19.692	5.128	100
Age of business (%)	600	2.875	0.344	1.869	3.584
Management costs (%)	600	8.467	3.241	3.119	17.512
Shareholding concentration (%)	600	58.862	19.594	25.815	100
Profitability (%)	600	8.603	10.441	-28.718	35.308
Development capacity (%)	600	-21.428	196.504	-1268.735	408.178
Solvency (%)	600	1.802	2.425	-3.744	10.485
Operating capacity (%)	600	3.714	4.612	-15.342	16.889
Type of enterprises	600	0.383	0.487	0	1
Type of two-job unity	600	0.35	0.477	0	1

### 4.3 Empirical analysis

This paper simultaneously uses its decomposition term pure technical efficiency and scale efficiency as explained variables to establish Tobit models (B) and (C). These models are used to further explore the influence of each explanatory variable on the specific ways in which the comprehensive efficiency. While the selection of comprehensive efficiency as an explained variable to establish Tobit model (A) can analyze the relationship between the influence of each explanatory variable on it, it is unable to clarify the specific path of the influence of each explanatory variable on the comprehensive efficiency. The Tobit model (A), (B), and (C) equations are displayed below, with the three-stage DEA model’s measurements of scale efficiency, comprehensive efficiency, and pure technical efficiency serving as the explained variables:

$$\begin{array}{l} Te_{it}=\beta_{0}+\beta_{1}Huma_{it}+\beta_{2}Age_{it}+\beta_{3}Ad\min_{it}+\beta_{4}Cr5_{it}\\ \;+\beta_{5}\mathrm{Pr}of_{it}+\beta_{6}Deve_{it}+\beta_{7}Debt_{it}+\beta_{8}Oper_{it}+\varepsilon_{it} \end{array}$$ (A)

$$\begin{array}{l} Pte_{it}=\beta_{0}+\beta_{1}Huma_{it}+\beta_{2}Age_{it}+\beta_{3}Ad\min_{it}+\beta_{4}Cr5_{it}\\ \;+\beta_{5}\mathrm{Pr}of_{it}+\beta_{6}Deve_{it}+\beta_{7}Debt_{it}+\beta_{8}Oper_{it}+\varepsilon_{it} \end{array}$$ (B)

$$\begin{array}{l} Se_{it}=\beta_{0}+\beta_{1}Huma_{it}+\beta_{2}Age_{it}+\beta_{3}Ad\min_{it}+\beta_{4}Cr5_{it}\\ \;+\beta_{5}\mathrm{Pr}of_{it}+\beta_{6}Deve_{it}+\beta_{7}Debt_{it}+\beta_{8}Oper_{it}+\varepsilon_{it} \end{array}$$ (C)

Where *Te*, *Pte* and *Se* represent comprehensive efficiency, pure technical efficiency and scale efficiency respectively, *β*_0_ is the constant term, *β*_*j*_(*j* = 1,2,⋯,10) is the regression coefficient of each variable, *i*(*i* = 1,2,⋯,60) is the number of firms ranked, *t*(*t* = 2013,2014,⋯,2022) represents the period, *ε*_*it*_ are the residual terms and satisfy *ε*_*i*_~*N*(0,*σ*^2^).

#### 4.3.1 Benchmark model estimation

The overall regression of the Tobit model is performed on models (A), (B), and (C), respectively, with the use of the stata17.0 software program. The outcomes are displayed in Table 11. The impulse response function is applied to simulate the dynamic influence of each variable on the technological innovation efficiency of textile and garment apparel listed enterprises in order to further analyze the dynamic influence process of management factors and financial factors on the technological innovation efficiency of enterprises. The result is the impulse response diagram, as illustrated in Fig 4.

**Table 11:** Tobit model overall regression.

Variable	Model (A)	Model (B)	Model (C)
Huma	0.00134*** (3.40)	0.00122** (3.13)	0.000657*** (3.51)
Age	-0.168*** (-6.19)	-0.19*** (-7.53)	-0.00646 (-0.52)
Admin	-0.00524** (-2.45)	-0.00323 (-1.62)	-0.00257** (-2.49)
Cr5	-0.00172*** (-3.29)	-0.000742 (-1.52)	-0.000794** (-3.13)
Prof	0.00185* (1.85)	0.00143 (1.54)	0.00154** (2.87)
Deve	-0.0000529** (-2.14)	-0.0000827*** (-3.45)	-0.00000774 (-0.56)
Debt	-0.00349* (-1.79)	-0.00123 (-0.69)	-0.00191* (-1.74)
Oper	0.00669** (3.00)	0.0036* (1.73)	0.00265** (2.19)
_cons	1.472*** (14.46)	1.52*** (16.05)	1.027*** (21.83)
Sigma_U	0.112*** (8.88)	0.115*** (8.86)	0.0317*** (7.09)
Sigma_E	0.0828*** (27.12)	0.0724*** (24.96)	0.0482*** (27.16)
*N*	600	600	600
Rho	0.646	0.715	0.302

Note: ***, **, and * indicate that the model regression coefficients are significant at the 1%, 5%, and 10% significance levels, respectively; parentheses are the Z-values corresponding to the regression coefficients.

**Fig 5:**
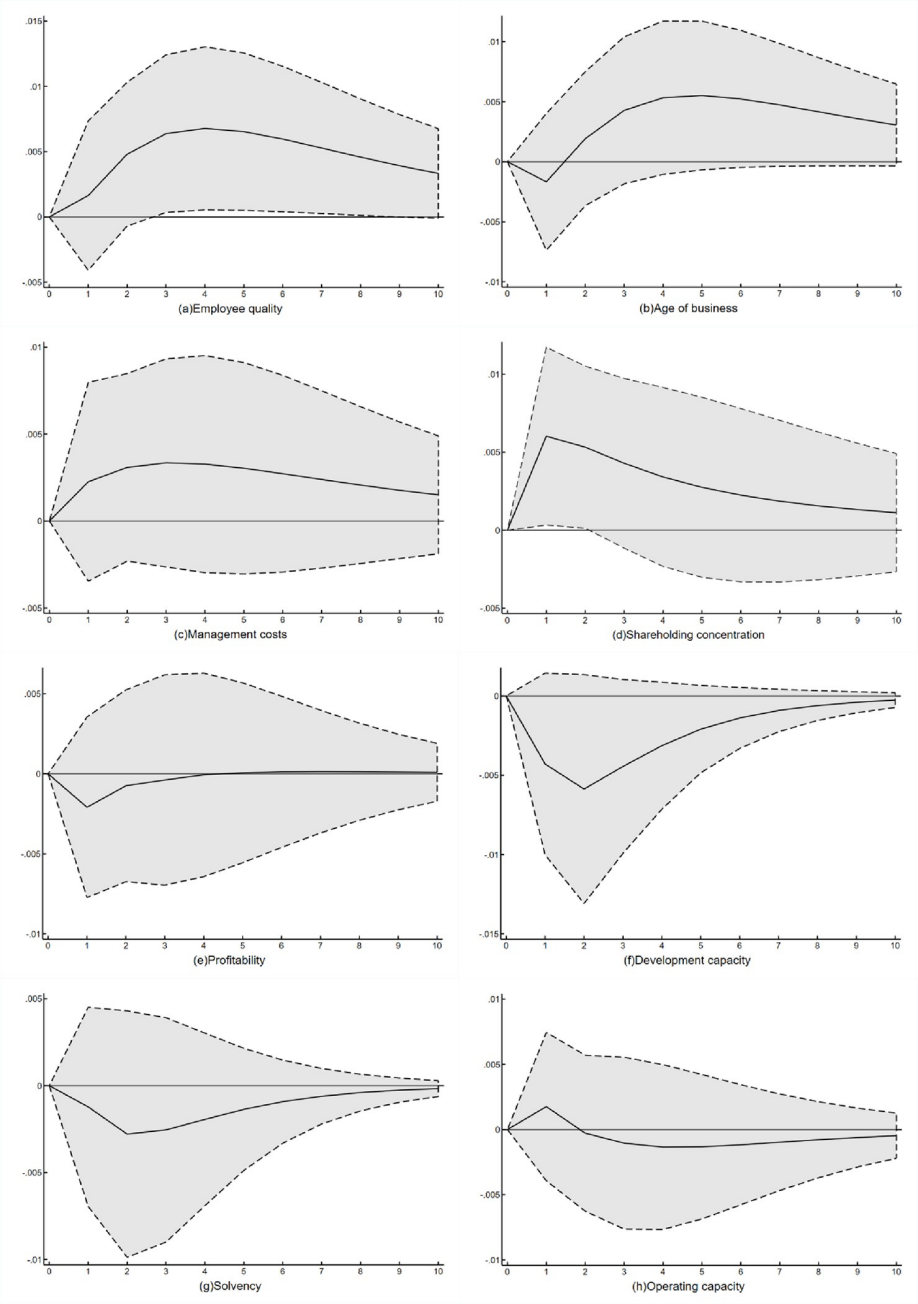
Impulse response graph.

Based on the results in Table 11 and Fig 5, the following conclusions can be drawn:

1. Employee quality, business age, management cost, and shareholding concentration are all correlated with comprehensive efficiency from the perspective of management factors, at least at the 5% significance level. This suggests that the enterprise’s management factors have a significant influence on comprehensive efficiency. The impulse response graph a shows that the impact of employee quality on the technological innovation efficiency of enterprises is effective over the long term and continues to show a positive correlation. According to this correlation, the more educated employees are in textile and clothing listed enterprises, the more favorable the development and utilization of technology, which in turn is conducive to enhancing the technological innovation efficiency of enterprises.
  The model (A) displays a negative correlation between the age of business, management costs, and shareholding concentration and comprehensive efficiency. However, the impulse response charts b, c, and d demonstrate a positive correlation between these three factors and the efficiency of technological innovation over time, with a significant impact that persists. This suggests that there is a degree of hysteresis between the three factors (the age of business, management costs, and shareholding concentration) and the enterprise’s technological innovation efficiency. In the short term, the three have side effects on the technological innovation efficiency of enterprises, but in the long term, the three have significant positive effects on the technological innovation efficiency of enterprises.
  According to the comparison of model (A) with model (B) and model (C), it can be seen that the quality of employees affects the comprehensive efficiency by influencing the dual factors of pure technical efficiency and scale efficiency, the age of the enterprise plays a role in the comprehensive efficiency by influencing the pure technical efficiency, and the management cost and the concentration of shareholding affects the comprehensive efficiency of the enterprise through the scale efficiency.
2. From the viewpoint of financial factors, profitability, development ability, solvency and operating capacity are all related to comprehensive efficiency at least at the 10% significance level. Furthermore, profitability and operating capacity are positively correlated with comprehensive efficiency, while development ability and solvency are negatively correlated, which is also consistent with theoretical expectations.

The higher the development capacity and solvency for textile and clothing listed enterprises, the more funds the enterprise must set aside as reserves from annual profits, which means that the enterprise’s investment in technology research and development must decrease. This is not conducive to the enhancement of technological innovation efficiency, as can be seen from the impulse response diagram f, g, which shows the negative impact of development capacity and solvency on the enterprise’s ability to achieve maximum value in technological innovation and has a long-term negative pull effect. However, from the impulse response diagram e, h, profitability and operating capacity in the long term on the enterprise’s technological innovation efficiency presents a negative effect, indicating that the enterprise should focus more on its overall business strategy than just its current state of affairs. If an enterprise’s profitability and operating capacity are higher, it means that its operating condition is better, the corresponding profit margin will be higher, and it can have sufficient funds to invest in technology development. This suggests enterprises should concentrate on long-term stable development in addition to the current economic climate.

When models (A), (B), and (C) are compared, it is clear that operating capacity influences comprehensive efficiency through its dual effects on pure technical efficiency and scale efficiency, profitability and solvency influence comprehensive efficiency through their effects on scale efficiency, and development ability influences the enterprise’s comprehensive efficiency through its effects on pure technical efficiency.

#### 4.3.2 Regression of firm heterogeneity

The existence of resource endowment and decision-making accuracy differences between enterprises will undoubtedly result in different technological innovation activities of enterprises in different contexts. For this reason, this study examines group regression from the perspectives of two-job unity and enterprise type, respectively, and examines how each influencing factor affects the role of integrated efficiency differences in the presence of enterprise heterogeneity. The regression results are displayed in Table 12.

**Table 12:** Tobit regression for firm heterogeneity.

Variable	Type of enterprises		Type of two-job unity	
	State-owned enterprises	Private enterprises	Two-job unity enterprises	Two-job non-unity enterprises
Huma	0.00119 (1.6)	0.00164*** (3.38)	0.0025*** (4.38)	0.0000235 (0.04)
Age	-0.233*** (-4.96)	-0.116*** (-3.57)	-0.179*** (-3.52)	-0.169*** (-5.22)
Admin	-0.012*** (-3.39)	0.000868 (0.32)	-0.015*** (-3.44)	-0.00194 (-0.78)
Cr5	-0.00128 (-1.07)	-0.000516 (-0.81)	-0.00276** (-3.06)	-0.0011* (-1.66)
Prof	-0.000946 (-0.45)	0.00309** (2.68)	0.00268 (1.62)	0.00158 (1.28)
Deve	-0.000068* (-1.66)	-0.0000453 (-1.41)	-0.0000334 (-0.86)	-0.0000443 (-1.42)
Debt	-0.00396 (-1.18)	-0.00231 (-0.96)	-0.00964** (-2.59)	-0.00126 (-0.56)
Oper	0.015** (3.12)	0.00323 (1.24)	0.0102** (2.66)	0.00397 (1.45)
_cons	1.729*** (9.79)	1.171*** (9.35)	1.624*** (8.03)	1.448*** (12.29)
Sigma_U	0.138*** (5.67)	0.0932*** (6.69)	0.123*** (5.12)	0.112*** (7.19)
Sigma_E	0.0858*** (15.73)	0.0792*** (22.09)	0.0822*** (14.98)	0.0796*** (22.61)
*N*	230	370	210	390
Rho	0.721	0.58	0.692	0.663

Note: ***, **, and * indicate that the model regression coefficients are significant at the 1%, 5%, and 10% significance levels, respectively; parentheses are the Z-values corresponding to the regression coefficients.

The technological innovation efficiency of state-owned and private enterprises is influenced by a variety of factors, as can be seen from a comparison of the enterprise type classification results. State-owned enterprises’ technological innovation efficiency is primarily influenced by their age, management costs, operating capacity, and capacity for development, whereas private enterprises’ technological innovation efficiency is primarily influenced by their employee quality, age, and profitability. Business age significantly influences both state-owned and private enterprises’ technological innovation efficiency, indicating that as enterprise establishment times increase, so does the corresponding technological innovation efficiency. However, the technological innovation efficiency of both types of enterprises is no longer significantly influenced by shareholding concentration, indicating that neither type’s equity (centralized or decentralized) can effectively affect the improvement of the enterprise’s technological innovation efficiency.

The technological innovation efficiency of the business responds significantly to the age of the business and shareholding concentration of the enterprise, regardless of whether the chairman and general manager of the enterprise are the same person or not. This means that as the number of years the business has been in operation increases and equity continues to be concentrated, the technological innovation efficiency of the business also increases significantly, regardless of whether the managers of the business are the same person or not. The response to the profitability and development capacity of financial factors is no longer significant for enterprises where the same person holds both positions. This is primarily because having the same person serve as both the general manager and the chairman of the board of directors increases the likelihood of an excessive concentration of power, which encourages managers to prioritize their own interests over the enterprise’s profitability and development potential. The technical innovation efficiency is no longer significant for all financial factors in businesses when the general manager and the chairman are not the same person. It is only meaningful for the age of the firm and the concentration of shareholding in the management factors.

### 5 Research findings and policy implications

This paper takes Chinese textile and garment listed enterprises as samples. First, the textile and garment industry’s technological innovation efficiency is evaluated against that of the manufacturing sector overall and each of its subsectors. This is done by measuring the technological innovation efficiency of listed textile and garment firms using a three-stage DEA technique. Second, utilizing the Malmquist index methodology, the technological innovation efficiency of listed textile and apparel companies was dynamically analyzed. This analysis was primarily focused on time series changes and enterprise heterogeneity. The impact of each influencing factor on the technological innovation efficiency of listed textile and garment enterprises is finally verified from the perspectives of financial and management factors, and examined in groups from the perspectives of enterprise type and two-job integration, all based on the Tobit model. The following are the study’s conclusions:

1. When viewed from a static perspective, the total technical innovation efficiency of textile and apparel companies between 2013 and 2022 exhibits a downward trend. In contrast to pure technical efficiency, average scale efficiency makes a greater contribution to the enhancement of the average comprehensive efficiency value of companies. Regarding enterprise types, state-owned businesses have an average comprehensive efficiency value that is higher than that of private businesses, while businesses with two-job unity each have an average comprehensive efficiency value that is higher than that of businesses with two-job non-unity. The textile and clothing industry’s technological innovation efficiency value is generally lower than that of the manufacturing industry as a whole and its various sub-sectors. This results in a significant reduction of the manufacturing industry’s total technological innovation efficiency value.
2. The dynamic analysis of listed textile and apparel firms’ technical innovation efficiency is based on the Malmquist index model. According to the time series change perspective, the total factor productivity of textile and clothing enterprises increased by 1.7% between 2013 and 2022; however, the overall level of development is low, and the fluctuation state displays a "W" type. The only years with increasing growth rates are 2014–2015, 2017–2018, 2019–2020, and 2021–2022, with the greatest year’s growth rate being just 7.3%. When looking at enterprise heterogeneity, the total factor productivity of both state-owned and private businesses is declining. State-owned businesses are declining by 1.9 percent, while private businesses are declining by 1.6 percent. This is not a significant difference between the two. When compared to the 1.5 percent decline in the total factor productivity of businesses with two-job non-unity, the two-job unity business decline is even higher, coming in at 2.1 percent.
3. Comprehensive efficiency is significantly impacted by both financial and management factors. In particular, management factors and comprehensive efficiency have a significant correlation, at least at the 5% level, suggesting that the management factors of enterprises have a significant impact on comprehensive efficiency. Among them, enterprise age, management cost, and equity concentration are all negatively correlated with comprehensive efficiency; however, employee quality is positively correlated with comprehensive efficiency and effective over the long term. The impulse response graph shows that all three of them are positively correlated with technological innovation efficiency over the long term, and the impact is still significant. At least at the 10% significance level, financial factors are related to overall efficiency. Development and debt servicing abilities are negatively correlated with overall efficiency, whereas profitability and operating ability are positively correlated with overall efficiency, in line with theoretical predictions.
4. According to the comparison of Tobit model (A), (B), and (C). Employee quality, operational capacity affects the combined efficiency by influencing both pure technical efficiency and scale efficiency. Firm age, development capability act on the combined efficiency by over affecting the pure technical efficiency. Management costs, equity concentration, profitability and solvency, on the other hand, affect the firm’s comprehensive efficiency through scale efficiency. When considering the heterogeneity of enterprises, the technical innovation efficiency of state-owned businesses is primarily influenced by their age, management costs, operational and development capabilities, and staff quality. In contrast, the technical innovation efficiency of private businesses is primarily determined by their age, profitability, and quality of personnel. The technological innovation efficiency of an enterprise is highly influenced by age and equity concentration, regardless of whether the person holding the positions of chairman and general manager is the same person. An enterprise with the same person in both positions is less likely to be affected by financial factors such as profitability and development capacity, and an enterprise with the same person in neither position is less likely to be affected by all financial factors.

Policy insights based on the above findings:

1. enhancing internal management and corporate governance. Organizational efficiency can be improved by enterprises through further reforming their corporate governance structure and streamlining decision-making and execution processes. This entails putting in place a strong internal control framework that works in tandem with incentives and disincentives in addition to enhancing management’s professional proficiency and strategic vision through HRD initiatives.
2. Improving the safeguarding of shareholders’ rights and interests and streamlining the shareholding structure. The shareholding structure of listed textile firms needs to be adjusted, the interests of major and small and medium-sized shareholders need to be balanced, and all shareholders’ rights and interests need to be fairly protected. In addition, a well-functioning oversight system can avert power abuse and advance the enterprise’s long-term growth, while a moderately concentrated ownership can aid in stabilizing the business plan of the organization.
3. Introducing the capital market to institutional investors. Establishing collaborative ties with professional investment institutions is recommended for firms. By leveraging their proficiency in capital operation, market analysis, and risk management, these institutions may offer steady financial support and strategic guidance to businesses. Through this engagement, businesses will not only be able to secure creative finance but also increase their credibility and transparency in the capital market.
4. Create an incentive program with a long lifespan to allow staff members to share in growth. Businesses can encourage employee creativity and loyalty by creating a variety of incentive programs (such as performance bonuses, equity incentives, and so on). It is recommended that more alluring long-term incentive mechanisms be implemented, particularly for important technical staff and managers, to achieve the shared growth objectives of employees and businesses.

### 6 Research limitations and perspectives

Although this paper maintains rigor in the research process, there are still some limitations, mainly including the following:

1. The sample selection’s limitations. This research only included a sample of Chinese Shanghai and Shenzhen A-share companies that were listed in the textile and apparel industry between 2013 and 2022. While this sample is representative, it does not include all relevant companies, particularly those that might not be listed on a stock market. This might restrict how broadly the findings can be applied.
2. Restrictions on data accessibility. The study’s data came from publicly available sources; however, some internal or sensitive data were not made available, which could have an effect on the model’s accuracy.
3. Methodological constraints and modeling. While the Tobit model and the three-stage DEA model perform better at eliminating the impacts of random mistakes and the environment, each statistical model has its own set of presumptions and assumptions, so it might not be able to fully account for all the variables that influence how efficiently technological innovation occurs.

The subsequent research should concentrate on the following areas in order to be able to more precisely quantify the effectiveness of technological innovation and its influencing factors:

1. Increasing the sample’s range. More businesses in more industries or geographical areas might be included in future research to improve the generalizability of the results and the breadth of the comparative study.
2. Analysis of longitudinal data. The dynamically shifting relationship between equity structure and enterprises’ technological innovation efficiency can be better understood by gathering longer time-series data for longitudinal study.
3. Comprehensive multimethod analysis. combining qualitative research techniques like case studies and interviews with quantitative analysis to produce more thorough findings.

## Supporting information

S1 Raw data (ZIP)
